# Biodegradability of Gel-Forming Superabsorbents for Soil Conditioning: Kinetic Assessment Based on CO_2_ Emissions

**DOI:** 10.3390/polym15173582

**Published:** 2023-08-29

**Authors:** Andrey V. Smagin, Nadezhda B. Sadovnikova, Elena A. Belyaeva, Christina V. Korchagina

**Affiliations:** 1 Soil Science Department and Eurasian Center for Food Security, Lomonosov Moscow State University, GSP-1, Leninskie Gory, Moscow 119991, Russia; nsadovnik@rambler.ru; 2 Institute of Forest Science, Russian Academy of Sciences (ILAN), 21, Sovetskaya, Moscow Region, Uspenskoe 143030, Russia; lllol871990@mail.ru (E.A.B.); korchagina@ilan.ras.ru (C.V.K.)

**Keywords:** synthetic polymer hydrogels, soil water superabsorbents, biodegradation, CO_2_ emission, kinetic curves, half-life, CO_2_ interfacial interactions, instrumental measurements, mathematical modeling

## Abstract

Quantification of the biodegradability of soil water superabsorbents is necessary for a reasonable prediction of their stability and functioning. A new methodological approach to assessing the biodegradability of these polymer materials has been implemented on the basis of PASCO (USA) instrumentation for continuous registration of kinetic CO_2_ emission curves in laboratory incubation experiments with various hydrogels, including the well-known trade brands Aquasorb, Zeba, and innovative Russian Aquapastus composites with an acrylic polymer matrix. Original kinetic models were proposed to describe different types of respiratory curves and calculate half-life indicators of the studied superabsorbents. Comparative analysis of the new approach with the assessment by biological oxygen demand revealed for the first time the significance of CO_2_ dissolution in the liquid phase of gel structures during their incubation. Experiments have shown a tenfold reduction in half-life up to 0.1–0.3 years for a priori non-biodegradable synthetic superabsorbents under the influence of compost extract. The incorporation of silver ions into Aquapastus innovative composites at a dose of 0.1% or 10 ppm in swollen gel structures effectively increases their stability, prolonging the half-life to 10 years and more, or almost twice the Western stability standard for polymer ameliorants.

## 1. Introduction

Gel-forming superabsorbents based on weakly crosslinked polyacrylamide, copolymers of acrylamide, and acrylic acid salts and their composites with mineral fillers (zeolites and phyllosilicates) and amphiphilic biopolymers from natural raw materials (peat, humates, and lignin), intercalated with ions of monovalent metals (potassium, sodium, ammonium, lithium, etc.), can be successfully used for water conservation in the soil by reducing unproductive losses due to evaporation and infiltration [1,2,3,4,5,6,7,8,9,10]. Small effective doses of these conditioners (0.05–0.5%), ten or even a hundred times lower compared to traditional soil ameliorants, ensure their high potential profitability not only in direct application but also in logistics [1,5,6,7,10]. Such doses can increase the water-retaining capacity of soils by 3–5 times, the range of available water content by 1.5–2 times, reduce physical evaporation by 1.3–3 times, and unproductive losses during infiltration up to 2–10 times [1,2,4,5,6,9,10]. Similar and even smaller doses of gel-forming polymeric materials strongly aggregate soil particles and protect them from water and wind erosion [8,9]. Along with the optimization of the water retention and soil structure, gel-forming polymers can be successfully used as agents for controlled release systems for pesticides and agrochemicals [4,5,6]. Intercalation of natural and synthetic biocides (heavy metal ions and nanoparticles, ammonium or sulfonium salts, synthetic fungicides, and antimicrobial peptides) in composite acrylic superabsorbents, as well as biomimetic technologies for the synthesis of polymethacrylates with a cationic and membranolytic biocidal effect, allow obtaining materials for environmentally friendly control of pathogens with low effective concentrations *E*_50_ of biocides near 10–100 ppm (soil) and 100–6000 ppb (water) [11,12,13].

However, most of these promising results are usually obtained in laboratory tests of polymer superabsorbents, whereas the real biologically and biochemically active soil environment can greatly limit their effectiveness. The most serious and yet neglected natural factor can be the biodegradation of synthetic polymeric materials in a real natural environment [13,14,15,16,17,18,19,20,21,22,23,24,25]. Our pioneering work [17], based on a thermodynamic analysis of the dynamics of water retention in coarse-textured soils under the influence of radiation-crosslinked polyacrylamide (PAA), revealed a significant (from 30 to 50% or more) reduction in the water capacity of the samples in the range of soil water pressures of 0–1000 kPa (centrifugation method) during a 6-month incubation experiment at different temperatures from 20 to 37 °C. Similar results were obtained for the Iranian acrylic superabsorbent Super AB, A-200 in field lysimetric experiments followed by thermodynamic analysis of water retention curves in the range of 0–1600 kPa (membrane press method) in a study [18]. Guided by the traditional point of view on the “non-biodegradability” of synthetic superabsorbents, the authors [18] did not associate the loss in water retention with biodegradation, explaining them by the indefinite “aging” of the material and the suppression of its swelling under the action of external (lithological) pressure or osmotic stress from soil electrolytes. Recently, however, more and more researchers are inclined to think about the conditionality of the traditional division of such superabsorbents into “biodegradable” (usually biopolymers–polysaccharides and composite gels based on them) and “non-biodegradable” (usually synthetic acrylic, acrylamide, acrylonitrile, and other polymers), emphasizing the potential importance of biodegradation in the behavior of both classes of gel-forming superabsorbents in soils [1,4,5,13,14,17,19]. Among the factors stimulating the biodegradation of stable polymers and composites, bioaugmentation and embedding of exoenzymes can have the strongest impact [23,24].

In this regard, the aim of our study was an instrumental assessment of the biodegradation of composite gel-forming soil conditioners with an acrylic polymer matrix in a fully automated incubation experiment based on PASCO equipment (PASCO Scientific, Roseville, CA, USA) for permanent monitoring of carbon dioxide emissions. This methodological study continues our previous development based on the kinetic assessment of biological oxygen demand (BOD) during long-term incubation of the same hydrogels using the VELP RESPIROMETRIC Sensor System 6 for Soils [13]. The main tasks included:

- Adaptation of new equipment to biodegradation studies of gel-forming soil conditioners;
- Obtaining and typing experimental kinetic curves of CO_2_ emission during gel incubation;
- Development of their physically based models for an adequate description of experimental data and calculation of basic biodegradability indicators in the form of half-lives of the studied materials;
- Analysis of the biodegradability of superabsorbents depending on their composition and incubation conditions;
- Methodological comparison of the results with previously obtained estimates based on BOD analysis and explanation of the reasons for their possible discrepancies.

In terms of scientific novelty, in addition to the new methodology, our experiments have revealed for the first time a strong contrast between the half-life values of pure hydrogels swollen in distilled water and gels of similar chemical composition that swelled in an aqueous medium with the addition of compost extract. It has also been experimentally confirmed that the introduction of silver ions into the polymer matrix effectively inhibits biodegradation and prolongs the half-life of composite gel-forming materials for soil conditioning. The new methodology and the results obtained on its basis can be useful for a wide range of specialists from chemical technologists to soil biologists, agricultural technologists, and landscape engineers engaged in the design of soils using innovative composite materials/superabsorbents to increase water retention, anti-erosion and anti-pathogenic soil protection, optimization of root nutrition, and productivity of cultivated plants.

## 2. Materials and Methods

The tested superabsorbents for soil conditioning were represented by two well-known brands, Aquasorb (SNF-group, [26]) and Zeba (UPL-group, [27]), as well as a line of innovative Russian materials, Aquapastus, produced by our patented technologies [6]. An obligatory hydrophilic component of the studied superabsorbents is a polymer matrix based on acrylamide (AA) and salts of acrylic acid (Ac), represented by potassium acrylate (Aquasorb and Zeba), and ammonium or sodium acrylates (Aquapasus) in various ratios of copolymers. Composite materials contained hydrophilic or amphiphilic copolymers or fillers of an acrylic polymer matrix, in particular starch (Zeba), waste from the biocatalytic production of polyacrylamide in the form of microbial cells and conglomerates (Aquapastus 11, or A11 further), and dispersed peat (Aquapastus 22, or A22 further). Two modifications of Aquapastus superabsorbents (A11-Ag and A22-Ag) contained 0.1% ionic silver additive as a biocide in the polymer matrix. The free-swell water absorption of the superabsorbents in distilled water ranged from 340 to 500 kg/kg. Detailed descriptions of the synthesis, composition, and results of preliminary laboratory testing of the Aquapastus composites for soil conditioning are presented in our previous articles [6,9] and patents (see Section 6 at the end of the article).

The hygroscopicity (*W_h_*), carbon content (*C*%), and *pH* data necessary to quantify the biodegradability of these materials (Table 1) were obtained by water thermo-desorption method [28], by coulometric titration method [29], and by the potentiometric method, respectively, using the AND MX-50 humidity analyzer (Japan, A&D, Tokyo), the AN-7529 C-analyzer (Gomel Plant of Measuring Devices, Gomel, Russia), and the combined EC/TDS/pH meter HANNA HI 98129 Combo (HANNA Instruments Deutschland GmbH, Fleringen, Germany).

Before incubation experiments, 1:100 swollen gel structures were obtained from 400 mg of air-dried superabsorbents. For this purpose, two types of liquid phase were used—pure distilled water and distilled water with an aqueous extract from compost consisting of rotting vegetables and fruits (potatoes, onions, apples, grapes, and oranges) mixed with soil humus [13]. The amount of carbon introduced with the compost extract (0.15–0.20 mg) was almost 1000 times less than the carbon content in the incubated gels and could not significantly affect the carbon balance of the experiment. The technique of closed incubation chambers was used by analogy with BOD analysis [13], where only the oxygen initially existing in the chamber (20.9% or 270–280 g/m^3^) was consumed during incubation. The initial concentration of CO_2_ (in the air of the laboratory) was in the range of 400–1200 ppm or 0.7–2.0 g/m^3^, and the final concentration at the end of the experiments did not exceed 28,000–76,000 ppm or 50–135 g/m^3^. The final oxygen concentration at the end of the experiment did not decrease below 13–18% (170–240 g/m^3^), i.e., the incubation conditions corresponded to aerobic biodegradation by analogy with the traditional BOD analysis [13].

**Figure A1:**
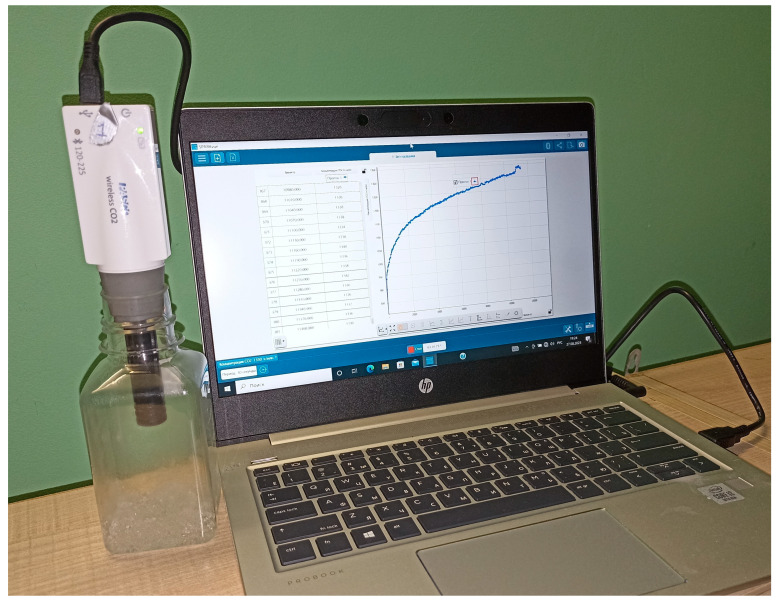
Installation for continuous monitoring of biodegradation in polymer hydrogels based on PASCO sensors.

The gel structures were placed in sealed incubation flasks with a free volume of 240 mL and a built-in wireless digital carbon dioxide sensor PS-3208 (PASCO Scientific, Roseville, CA, USA [30]) for continuous monitoring of CO_2_ emission during the incubation experiment. Closed flasks with gels and PS-3208 sensors tuned to take CO_2_ readings every 5 min were installed in a BINDER ED023-230V thermostat (BINDER GmbH, Tuttlingen, Germany) and exposed for 24–72 h at a constant temperature of 30 °C, optimal for aerobic biodegradation. At the end of the experiment, the sensor was connected to a computer via a USB port and using PASCO SPARKvue 4.0.0 software (PASCO Scientific, Roseville, CA, USA [30]), CO_2_ data were read in real time from the sensor’s memory and exported to MS Excel, Microsoft Office 2016 (Microsoft, Redmond, DC, USA), for further mathematical and statistical processing, as well as the preparation of illustrative material (see Figure A1 in Appendix A). The incubated materials (gel structures) in a closed vial were quickly (within 2 min) heated up to 75–80 °C in a microwave oven for rapid degassing of CO_2_ potentially dissolved in the liquid phase of the gel, according to [21]. Its volume ratio was determined directly in the vial using a portable gas analyzer OXYBABY M+ O_2_/CO_2_ (WITT-Gasetechnik, Witten, Germany).

The approximation of experimental CO_2_ data by the new models of incubation CO_2_ kinetics proposed in the study with the necessary statistical processing was carried out in S-Plot 11 (Systat Software GmbH, Erkrath, Germany) computer software using the nonlinear regression package “Regression Wizard”. Comparative statistical processing of the results with ANOVA was carried out in the R 3.5.3 program (RStudio PBC, Boston, MA, USA).

## 3. Results

### 3.1. Respirometric Curves and Their Description

**Figure 1:**
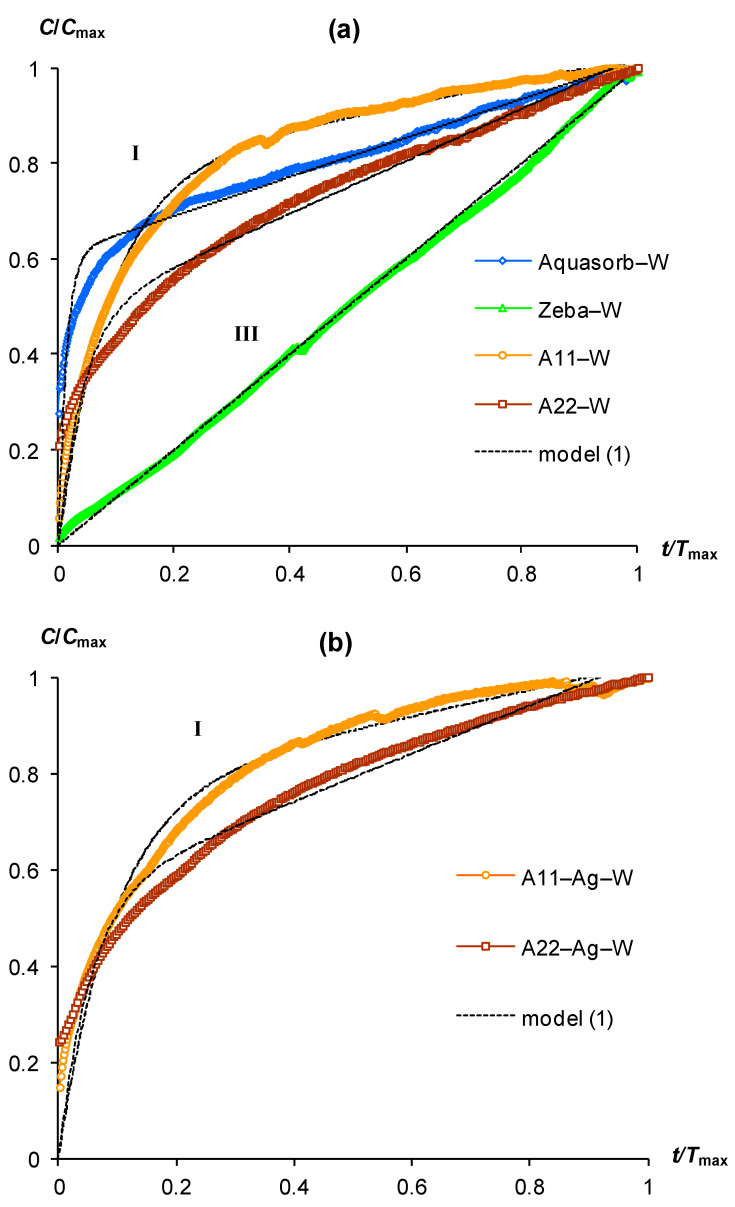
Scaling kinetic curves of CO_2_ emission in pure gels swollen in distilled water (W) and their approximation by model (1): (**a**) gels without biocides; (**b**) gels with ionic silver in a polymer matrix; **I**, **III**—types of kinetic curve shapes (see text).

**Figure 2:**
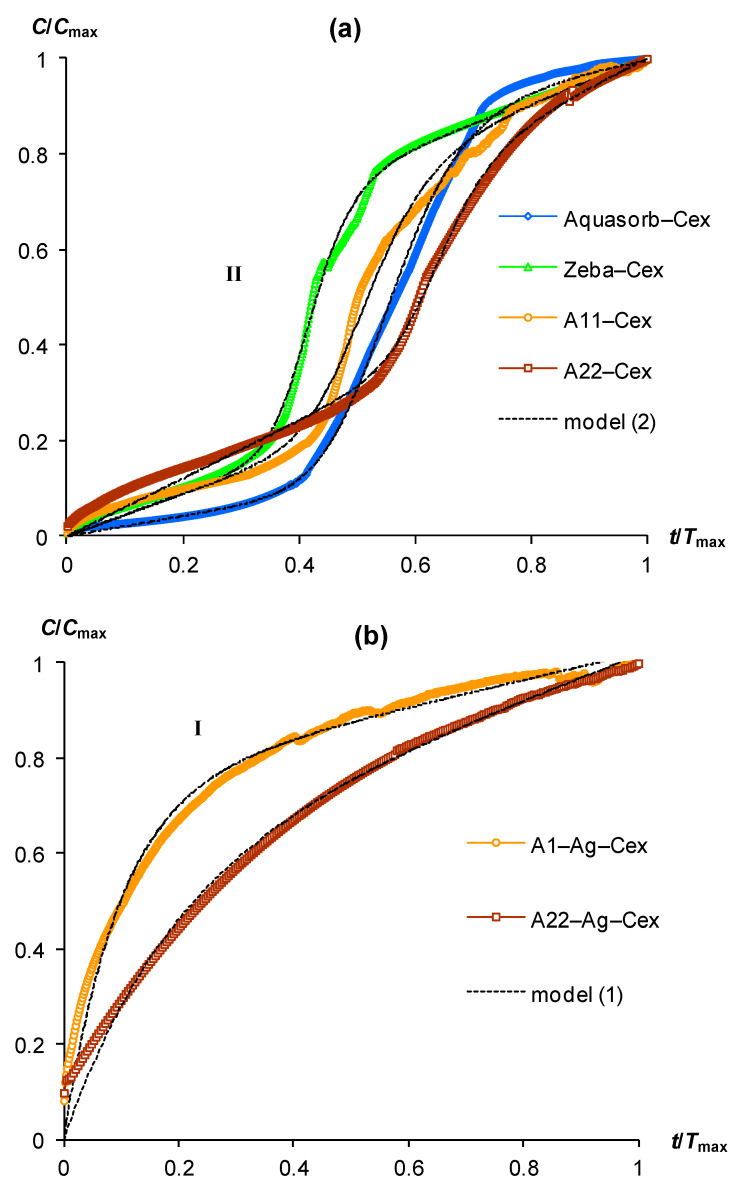
Scaling kinetic curves of CO_2_ emission in gels with additives of compost extract (Cex) in the liquid phase and their approximation by models (1) and (2): (**a**) gels without biocides; (**b**) gels with ionic silver in a polymer matrix; **I**, **II**—types of kinetic curve shapes (see text).

Figure 1 and Figure 2 represent typical kinetic curves of CO_2_ emission during incubation of gel-forming soil conditioners. For convenience, we performed a preliminary scaling of the experimental data through their normalization by the maximum values of the CO_2_ content (*C*_max_) and the duration of the experiment (*T*_max_). All kinetic curves could be divided into three types. The most common is type (I), which is a convex curve with a rapid increase in CO_2_ concentration at the initial stage of incubation and a subsequent gradual deceleration, reaching a constant (quasi-linear) small increase at the end of the experiment. This form can be explained by the inhibition of the rate of biodegradation by a lack of oxygen when it is consumed in an incubator closed for gas exchange [13,31,32]. Type (II) is characterized by a more complex shape of kinetic curves with a change from a concave section to a convex one during CO_2_ accumulation. The initial phase with a slow rate of CO_2_ growth is often interpreted as a lag phase in the development of a microbial population that decomposes the organic substrate [32]. The lag phase is followed by active growth of the colony with rapid destruction of the organic substrate and CO_2_ emission. Oxygen limitation, and possibly simultaneous inhibition of microorganisms by excess CO_2_, slow down their growth and biodegradation of the substrate, so the kinetic curve acquires a convex shape with a quasi-linear small increase in CO_2_ concentration at the final stage of the experiment, by analogy with curves of type (I). The rarest is type (III), which is characterized by a constant (quasi-linear) increase in the CO_2_ content in the incubator throughout the entire experiment. It can be explained both by the short duration of the experiment and by the susceptibility to biodegradation of the studied organic substrate with intense release of CO_2_ without any significant effect of its inhibition.

Figure 1 (upper part) represents the scaling kinetic curves of CO_2_ emission for gel-forming soil conditioners, pre-swollen to a water content of 100 g/g in distilled water (hereinafter referred to as “pure gels”). All hydrogels, except for the Zeba composite material, were characterized by convex curves of type (I) with a rapid increase in CO_2_ content (up to 60–80% of the maximum) at the initial stages of incubation (0.2–0.4*T*_max_) and a subsequent rather slow stage of steady biodegradation with an almost constant (quasi-linear) increase in CO_2_ content. The fastest transition to the quasi-equilibrium stage was observed for the Aquasorb gel (<0.2*T*_max_). The kinetic curve for the Zeba material belonged to type (III) and was characterized by a constant increase in CO_2_ throughout the experiment. The introduction of biocides in the form of silver ions into the polymer matrix of innovative materials A11-Ag and A22-Ag did not significantly affect the shape of the kinetic curves of CO_2_ emission, which became only slightly flatter, i.e., with a slower approach to the quasi-linear region (Figure 1, lower part).

Sharp changes in the kinetic curves of CO_2_ emission occurred for variants of hydrogels swelling in distilled water with the addition of compost extract (Figure 2a). This treatment changed the shape of kinetic curves from type (I) to type (II). Without exception, all kinetic curves of gel-forming soil conditioners have a lag phase segment with a duration from 0.3 to 0.4 *T*_max_. The intensity of CO_2_ emission in this area did not exceed 0.2 *C*_max_ (Figure 2a). Subsequently, the lag phase changed to a stage of very rapid growth (CO_2_ generation) with a maximum relative slope of the curves near 3/2 in the interval 0.4–0.6 (up to 0.8) *T*_max_. This stage changed to a quasi-linear section at the final phase of the incubation experiment. The introduction of silver ions into the polymer matrix of the A11-Ag and A22-Ag composites was reflected in the kinetic patterns of their biodegradation by changing the type of curve from (II) to (I) (Figure 2b). Therefore, for these biocide-embedded materials and incubation conditions involving swelling of the gels in pure distilled water or with the addition of compost extract did not affect the shape of the kinetic curve belonging to type (I) in both cases.

### 3.2. Mathematical Modeling of Respirometric Curves

All obtained types of biodegradation kinetic curves are known in the specialized literature, where numerous empirical models are proposed for their approximation [31,33]. In particular, convex curves of type (1) for the main nonlinear section are well fitted by the Larson model [31] in the form: *C*(*t*) = *K*(1 − exp(−*kt*)), where *K* is the maximum concentration of emitted CO_2_ or the maximum BOD, and *k* is the kinetic constant. This model, as shown in [13], follows from first-order biological kinetics under oxygen-limited biodegradation. The sigma shape (type II) is usually explained by second-order kinetics with the well-known Verhulst–Pearl model [34] and its various modifications applied to the process of microbial biodegradation of organic materials. The analytical solution of this model in the form: *C*(*t*) = *K*/(1 + Qexp(−*rt*)) (*K*, *Q*, and *r* are empirical constants) was preliminarily tested for the adequacy of fitting experimental data for curves of type (II). However, both known models could not adequately describe the entire range of experimental curves, including the finite quasi-linear section. In addition, a more suitable equation was found for curves of the sigmoid type (II). As a result, for scaling kinetic curves, we proposed the following two models with the best fitting results.

For curves of type (I) with a convex shape followed by a quasi-linear section:

(1) $$y_{\left(x\right)}=a\left(1-\exp(-b\cdot x)\right)+(1-a)x,$$

where y = *C*/*C*_max_ and *x* = *t*/*T*_max_ are dimensionless variables of CO_2_ concentration and experiment time; *a* = *K*/*C*_max_ and *b* = *T*_max_*k* are dimensionless concentration and kinetic parameters of the model.

For sigma-shaped curves of type (II) with a quasi-linear finite section:

(2) $$y_{\left(x\right)}=\frac{a}{1+{\left(\frac{x}{x_{0}}\right)}^{-r}}+(1-a)x$$

where *a* = *K*/*C*_max_, *x*_0_, and *r* are dimensionless concentration and kinetic constants. The first term of Equation (2) follows from the known Moser model [35], which is more complex than the second-order kinetics, and assumes a power–law relationship of the kinetic constant with the concentration of the biodegradable substrate. The analytical solution of similar models with delayed biodegradation kinetics gives a kind of sigmoid in the form *C*(*t*) = *Kt^r^*/(*Q* + *t^r^*), which at *t* ≠ 0 corresponds to the first term of our Equation (2).

Almost all studied gel-forming soil conditioners were characterized in incubation experiments by kinetic curves of type (I) or (II). The only exception was the Zeba composite material, which included an easily decomposing component in the form of starch. Its kinetic curve was represented by a linear dependence during the entire experiment (Figure 1a, line III). The approximation parameters of experimental data for pure gels and composites with silver by model (1) varied in the ranges 0.5 < *a* < 0.8 and 10 < *b* < 69, except for the Zeba material, where the use of model (1) was possible only at very low values *a*, *b* of the order of 10^−3^ (Table 2). All parameters were statistically significant at *p*-values in the range of 0.0013–0.0001, which, along with high coefficients of determination (R^2^ = 0.955–0.999) and small standard errors of estimate (s = 0.01–0.04), indicated the adequacy of the new model (1). Approximation of experimental curves of type (II) by the new model (2) also showed its high adequacy (R^2^ = 0.996–0.998, s = 0.01–0.02) with statistically significant parameters at the *p*-value < 0.0001, varying in the ranges: 0.4 ≤ *a* < 0.8; 9 < *r* <14; 0.4 < *x*_0_ < 0.7 (Table 2). Thus, both proposed models successfully fitted the experimental data for different types of kinetic curves in incubation experiment with gel-forming superabsorbents based on the new PASCO respirometric equipment.

### 3.3. Calculated Indicators of Biodegradability of Superabsorbents (Half-Lives)

The new models (1) and (2) are conveniently used not only to describe the kinetic curves of CO_2_ dynamics in incubation experiments but also to assess the biodegradability of the studied gel-forming materials. Traditionally, an indicator of the intensity of biodegradation is the half-life (*T*_0.5_) of organic matter or the time of decomposition of half of its initial amount [32]. In the first (linear) approximation, the half-life can be calculated from the percentage of decomposition (mass loss) of organic material (*D*%) for a known incubation time (*T_D_*) using the following formula [21]:

(3) $$T_{0.5}=T_{D}\frac{ln(2)}{\left[ln(100)-ln(100-D\%)\right]}$$

If the dimensionless index (a) in models (1) and (2) is estimated from the kinetic curves, it is easy to convert it into the specific rate of carbon dioxide emission (*A*, [gCO_2_/(m^3^day)]) at the last (quasi-linear) stage of biodegradation with a constant intensity, established after the development of the microbial population of destructor organisms:

(4) $$A=\frac{24\;(1-a)K}{T_{\max}}$$

where *K* (gCO_2_/m^3^) and *T*_max_ (hr) are the normalizing characteristics of the maximum carbon dioxide content and the duration of the incubation experiment, and 24 is the conversion factor of hours per day. The *K* value (gCO_2_/m^3^) is estimated from the Clapeyron–Mendeleev law according to the formula [21]:

(5) $$K=\frac{X_{\mathrm{ppm}}PM}{10^{6}RT},$$

where *X*_ppm_ is the maximum volumetric content of CO_2_ in the incubator determined by the PASCO gas analyzer, expressed in ppm, *P* (Pa) is atmospheric pressure, *T* (K) is the incubation temperature, *R* = 8.314 J/(mol K) is the universal gas constant, and *M* = 44 g/mol is the molar mass of carbon dioxide.

In this case, the percentage of degradation of the material at the established slow stage of biodegradation is given by:

(6) $$D\%=\frac{12}{44}\frac{A\cdot V\cdot 100\cdot \;(100+W_{h})}{m_{0}C_{\mathrm{org}}\%},$$

where *V* (m^3^) is the volume of the air space of the incubator, *C*_org_% is the percentage of organic carbon in the incubated material, *m*_0_ (g) is the weight of the air-dried sample of the material, *W_h_* (%) is the percentage of hygroscopic moisture in it, and 12*/*44 is a conversion factor that takes into account the mass fraction of carbon in CO_2_.

Substituting the value of *D*% into Equation (3) and taking into account that the parameter *T_D_* = 1 day, it is easy to calculate the half-life of the material. Note that taking into account hygroscopic water content in the calculations of *D*% and, respectively, *T*_0.5_ is extremely important for superabsorbents, since they are highly hygroscopic materials with *W_h_* values of 30% or more [28].

Along with the above estimation by models (8) and (9), we used additional options for determining *T*_0.5_ in the form of a potential range of this value *T*_0.5_^min^–*T*_0.5_^fin^. The lower boundary of this range (*T*_0.5_^min^) was determined by the mass of CO_2_ released over the entire period of the experiment, including the initial fast stage, which gives the following formula for calculating the percentage of decomposition:

(7) $$D\%=\frac{12}{44}\frac{\left(C_{\max}-C_{0})\cdot V\cdot 100\cdot \;(100+W_{h}\right)}{m_{0}C_{\mathrm{org}}\%},$$

where *C*_0_ and *C*_max_ (gCO_2_/m^3^) are the initial (atmospheric) and final (maximum) concentration of CO_2_ in the incubator. Substituting the value *D*% determined by Equation (7), along with the value of the experiment time *T_D_* = *T*_max_, into Equation (3) makes it possible to estimate *T*_0.5_^min^.

The upper boundary of the considered range (*T*_0.5_^fin^) is determined by direct linear approximation of the experimental data *C*(*t*) at the final (quasi-linear) stage of the experiment with the slowest emission rate by the straight-line equation:

(8) $$C_{\left(t\right)}=C_{K}+d\cdot t,$$

where *C_K_* (gCO_2_/m^3^) and *d* (gCO_2_/(m^3^h)) are approximation parameters. Substituting the value 24*d* instead of *A* into Equation (6) gives the minimum possible estimate of *D*% for the incubation experiment and, respectively, the maximum value of *T*_0.5_^fin^ according to Equation (3).

Note that all the discussed regularities and calculations were obtained for scaling curves reflecting relative changes in the biodegradation kinetics of the studied superabsorbents (Figure 1 and Figure 2). The absolute values of CO_2_ emission intensities and biodegradation rates of the studied materials are estimated by the corresponding normalizing indicators *C*_max_ and *T*_max_, as well as the indicators of half-lives calculated by the parameters of models (1) and (2) using theoretical Equations (3)–(8) (Table 3).

The *C*_max_ value varied from 1.8 to 135 gCO_2_/m^3^ and was the highest in experiments with the addition of a compost extract stimulating biodegradation. However, after thermo-desorption of potentially bound CO_2_ in the gel, according to [21], its concentration increased significantly, reaching 4.8–189 g/m^3^ (column *C*_t_ in Table 3). The time of experiments usually did not exceed three days (*T*_max_ = 60–72 h), which favorably distinguishes the new methodology from BOD analysis, where a similar assessment of biodegradation requires incubation of gel-forming soil conditioners for 1–3 months [13].

The half-lives of the studied superabsorbents varied widely from 0.1–0.2 to 10–14 years (Table 3). The highest values of *T*_0.5_, reflecting the maximum resistance to biodegradation, as expected, were found in the innovative A11-Ag and A22-Ag composites with silver biocides. The range of *T*_0.5_^min^–*T*_0.5_*^f^*^in^ estimates was 3.2–10.9 and 6.6–13.9 years, and the calculation using model (1) gave *T*_0.5_ = 13.8 *±* 0.5 and 12.3 *±* 0.3 years for A11-Ag and A22-Ag gels, respectively. These characteristics significantly (2–4 times or more) exceeded the half-lives for materials without biocidal additives, both in the original prototypes A11 and A22 and in the similar Western brands Aquasorb and Zeba. Among them, the Zeba composite material based on polyacrylamide and starch had the minimum stability (*T*_0.5_ ≈ 0.6 years), and the A22 composite with an acrylic matrix filled with dispersed peat had the maximum (*T*_0.5_ = 3.5–6.1 years). Materials A11 and Aquasorb occupied an intermediate position with *T*_0.5_ characteristics of 0.7–2.7 and 1.9–3.5 years, respectively.

The addition of compost extract to the liquid phase for the swelling of gels sharply increases the intensity of their biodegradation. The *T*_0.5_ values of gels decrease to 0.1–0.3 (0.6) years, i.e., by 6–10 times or more compared to pure samples. This result seems to be extremely important from a technological point of view, since it calls into question the traditional notions of high resistance to biodegradation of synthetic polymer superabsorbents in real soil conditions, where natural solid-phase components and solutions with destructor organisms and exoenzymes are present instead of almost sterile distilled water. Obviously, the reduction of the actual working life and functionality of rather expensive soil conditioners from several years to 0.5–1 year may well be the reason for refusing to use them due to lack of profitability. In this regard, technological developments of composite materials combining the properties of superabsorbents with reduced biodegradability are promising for soil conditioning [10].

For example, the introduction of silver ions into the polymer matrix of composite acrylic gels, which was originally proposed to combat potato pathogens, including phytophthora [6], simultaneously enhances their resistance to biodegradation. The *T*_0.5_ values for innovative A11-Ag and A22-Ag composites swollen in a liquid with compost extract increased to 2.4–3.4 years, or 5–10 times, actually returning to such materials the stability inherent in the initially “pure” state of swelling in a distillate (Table 3). However, the question of the optimal doses of biocides in innovative superabsorbents is still open. The used dose of 0.1% Ag in a polymer matrix or 10 ppm Ag in a swollen gel structure appears to be quite effective. However, previous half-life calculations did not take into account the amount of CO_2_ potentially immobilized in the gel structure. If we use the total amount of CO_2_, including CO_2_ immobilized in the gel structure, i.e.*,* the value of *C*_t_ instead of *C*_max_ in Equations (3)–(7), the half-life values decrease by 1.5–2 (up to 3) times (compare columns *T*_0.5_*^mod^* and *T*_0.5_*^term^* in Table 3). In particular, the half-life of innovative superabsorbents A11-Ag and A22-Ag swollen in water with compost extract is reduced by two times to values of 1.2–1.7 years with this method of evaluation. Such low half-life values for soil conditioners may be unacceptable from a technological point of view, and higher doses of biocides will be required to improve their resistance to biodegradation.

Statistical data processing confirmed the obtained results of comparing the biodegradability of different gels depending on the factors of their composition and incubation conditions (with and without compost extract). Preliminary testing of the entire CO_2_ data array (more than 8000 measurements) and the calculated half-life indicators (more than 60) revealed the problem of referring both data samples to a normal distribution (normality test) with uniform dispersion, since the Shapiro–Wilk, Bartlett, and Levene tests were not passed. Therefore, along with the standard ANOVA, we also used nonparametric ANOVA to compare the results of biodegradability of different superabsorbents (Table 4).

Both variants of the analysis confirmed the statistically significant (*p*-values = 1.9 × 10^−17^ and 9.9 × 10^−8^ for the Fisher and Kruskal–Wallis criteria less than the commonly used critical *p*-value = 0.05) effect of the composition factor of gel structures on their biodegradability. Post hoc group analysis by both methods showed statistically significant differences for the compared gels (highlighted in red in Table 4), the regularities outlined above, i.e.*,* differences in the half-lives for gels pre-swollen in pure distilled water and with the addition of compost extract. Moreover, statistically significant differences were found for superabsorbents with and without biocide. Innovative composites A22, A22-Ag, and A11-Ag were characterized by maximum differences from other studied superabsorbents, having statistically significantly the largest half-lives, both in parametric and nonparametric ANOVA (LSD and Newman–Keuls tests, respectively). Summarizing the experimental part, we especially note the most technologically important results on the enhancement of the potential biodegradability of synthetic superabsorbents under the influence of compost extract and on the biocidal effectiveness of silver ions in the polymer matrix of these materials.

## 4. Discussion

### 4.1. Comparison with Known Data and Discussion on the Biodegradability of Synthetic Superabsorbents

Our results partially clarify the existing rather contradictory information about the biodegradability of synthetic superabsorbents. It is traditionally believed that synthetic polymer hydrogels are very resistant to biodegradation and can be classified as “non-biodegradable”, in contrast to well-degradable “environmentally friendly” or “biodegradable” polysaccharide gels based on starch, cellulose, chitosan, and other biopolymers of natural origin [1,4,5,7,12,14,15]. At the same time, it is obvious that polymers that are completely resistant to degradation do not exist, and the question is only in characterizing the rate of their decomposition (characteristic lifetime) under certain conditions [32]. Formally, this issue is resolved in the Western examination of polymeric materials by two standards of biodegradability: European EN 13432 and American ASTM 6400 [36,37,38]. For the American standard, a material that decomposes by 60% in 180 days is considered biodegradable, and for a more stringent European standard, such a threshold is decomposition by 90% [36,37,38]. Using Equation (3), it is easy to determine that the conditional boundary for dividing materials into “biodegradable” and “non-biodegradable” according to American and European standards will be their “standard” half-lives of 136 and 54 days or 0.15–0.37 years, respectively.

Comparing our experimental data (Table 3) with these criteria, it is easy to make sure that pure acrylic gels swelling in distilled water are indeed “non-biodegradable”, since their half-lives of 2–3.5 years or more significantly exceed the limits of biodegradability standards in 0.15–0.37 years. In addition, only the Zeba composite material with an easily degradable biopolymer component in the form of starch and the corresponding characteristic *T*_0.5_ = 0.11–0.24 years can be conditionally classified as “biodegradable”, especially according to the American standard. This information is consistent with most of the known data on the degradation of acrylic superabsorbents [7,39,40]. Thus, Lenz et al. [39] reported a 10% destruction of polyacrylamide (PAA) per year, which in terms of the half-life according to Equation (3) gives *T*_0.5_ = 6.5 years. Close estimates of *T*_0.5_ = 5–7 years for synthetic polymeric superabsorbents are given in [7]. In [40], the degree of PAA mineralization in the soil is estimated at 22.5% for 2 years or a half-life of 5.5 years.

At the same time, a number of publications contain information about the possibility of rapid decomposition of acrylamide, PAA, and other acrylic gels with half-lives significantly lower than the EN 13432 and ASTM 6400 standards [14,19,20,22,41,42,43,44]. The half-lives of acrylamide in soils and water according to data [14,41] recalculated by Equation (3) do not exceed 3–14 days. The kinetic constants of PAA biodegradation from [19] give the values of its half-life from 37 to 80 days in the experimental temperature range of 25–37 °C. The study [20] provides data on the biodegradation of a superabsorbent based on copolymers of acrylamide and potassium acrylate by soil bacteria, from which it follows that the half-lives of such a hydrogel vary from 0.13 to 1.31 years. The authors [20] also mention a rather strong (about 25%) loss of water-holding capacity of this material during the 8-month biodegradation, which is consistent with some other similar data, including our own studies [13] (see Introduction).

Comparison of our results for incubation of superabsorbents alone and with the addition of compost extract (Table 3 and Table 4) clarifies the most likely reason for the strong variation in literature data for the rate of biodegradation of acrylic gel-forming polymers. The presence of biodestructor organisms and their enzymes necessary for biodegradation causes a sharp increase in the intensity of decomposition of these synthetic polymers, which are a priori stable due to chemical crosslinking and high molecular weight. This conclusion is also confirmed by recent studies [25] on the assessment of the biodegradability of gel-forming soil conditioners depending on the synthesis parameters (degree of swelling and chemical composition), as well as environmental conditions. If the untreated control (pure gels) in [25] remained stable for 8 days of the incubation experiment (weight loss up to 3–5%, statistically not significantly different from zero), contact with forest soil led to losses of 30–40% and with agricultural soil to losses of 60–70% of polymer composites due to biodegradation. Such losses in terms of half-life are *T*_0.5_ from 6 to 15 days, or significantly less than 1 month. The main factor in the significant increase in biodegradation after direct contact with soils and plants or after the addition of aqueous extracts from them, of course, is the presence of soil microorganisms/biodegraders and exoenzymes. Additionally, we can assume the priming effect, which is well known in soil biochemistry [45]. In this regard, the traditional division of superabsorbents into “biodegradable” biopolymers and “non-biodegradable” synthetic polymers becomes very conditional [13]. Particularly strong biodegradation damage to polymer hydrogels is expected in arid irrigated agriculture, since polymers intended for water retention always contain water available for microbes, and high (up to 40–50 °C) temperatures exponentially increase their biodegradative activity for polymers in accordance with the van’t Hoff rule [17,24].

Obviously, for soil gel conditioners, rapid biodegradation with loss of functionality is a serious reason for their potential unprofitability. The authors in [46] cite a German quality standard requiring a degradation of no more than 20% over a 2-year period (half-life of about 6 years) for synthetic ameliorant polymers in soil. Table 3 shows that, in reality, none of the biocide-free acrylic composites, including the well-known Aquasorb brand, pass this rigorous test. A similar result is obtained from the analysis of published information [7,13,15,16,17,21,25,47], where no more than 10% of superabsorbents meet this standard, and 60% of materials lose half of their mass (and, therefore, functionality) within 1 year of use. This serious problem poses the challenge of further improvement of gel-forming composite materials for soil conditioning with higher resistance to biological degradation.

A promising way to reduce the biodegradability of polymeric materials can be the introduction of biocidal components into their composition [21,46]. In this study and in previous works [6,9,10,13,21], we, probably for the first time for soil conditioners, proposed the use of silver ions and nanoparticles as biocides. Known similar developments with silver biocides in gels mainly concern medical preparations and antiseptics [12,48]. Silver biocides effectively increase the resistance of hydrogels to biodegradation, which is confirmed by both previously published data for laboratory [9,13,21] and field [6,10] tests as well as new results based on PASCO equipment (Table 3 and Table 4). Our results are in full agreement with the data [49,50,51,52] reporting the high efficiency of silver biocides at relatively low doses (1–100 ppm). When the innovative gels A11-Ag and A22-Ag are swollen up to 100 g/g, the initial silver content of 0.1% will be equivalent to its working concentration in the gel structure of 10 ppm. This dose, as can be seen from Table 3, is enough to give the soil conditioner the stability required by the German quality standard [46] (*T*_0.5_ about 6 years), and at the same time, it is not dangerous for plants and soil zoocenosis, including earthworms, with effective concentrations of biocidal suppression of 250–500 ppm and more [21,51,53]. However, due to the relatively high cost of silver, future research should be directed to finding alternative biocidal additives, for example, based on copper, antimony, ammonium, phosphonium or sulfonium salts, and titanium oxide, as well as organic antimicrobials [1,44,54].

### 4.2. Methodological Aspects of the Study (Comparison of CO_2_ and BOD Analysis Data)

**Figure 3:**
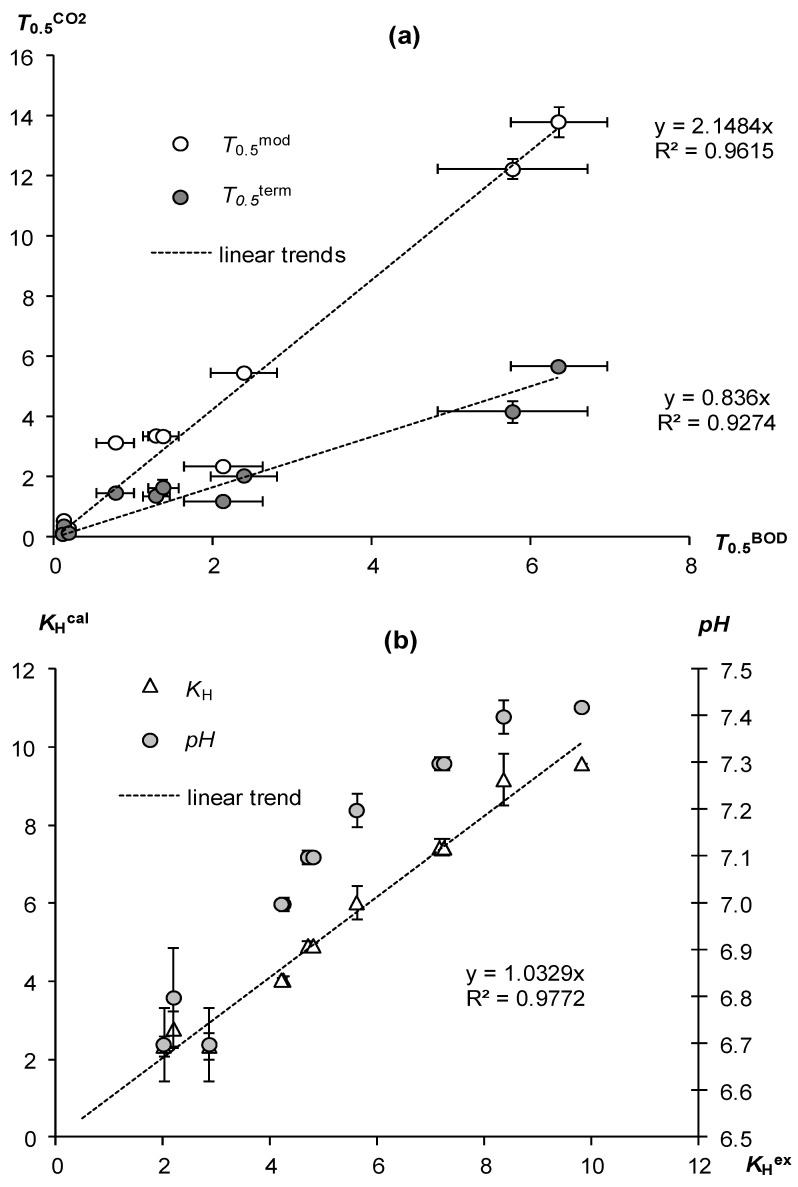
Discrepancy between CO_2_ and BOD estimation of superabsorbents’ biodegradability and its possible cause. (**a**) Correlation of the half-lives of superabsorbents estimated by BOD (*T*_0.5_^BOD^), by normal CO_2_ emission (*T*_0.5_^mod^), and by CO_2_ emission taking into account CO_2_ immobilization in the gel structure (*T*_0.5_^term^); (**b**) comparison of the experimental and calculated results by the model (10) Henry constants for the dissolution of CO_2_ in the liquid phase of the gel.

Our previous methodological study [13] used biological oxygen demand (BOD) to analyze the biodegradability of superabsorbents during their laboratory incubation. In this regard, it is interesting to compare the data obtained by two methods for laboratory evaluation of biodegradability in the studied polymeric materials. Figure 3a shows the differences in the estimates of the biodegradability of superabsorbents by BOD and by CO_2_ emission. Conventional analysis involves only monitoring CO_2_ emissions in the gas space of the incubator. In this case, as can be seen from the upper graph of Figure 3a, the CO_2_ estimate gives a significant excess compared to the BOD estimate. Both T_0.5_^BOD^ and T_0.5_^mod^ half-life indicators are well correlated (Pearson*’*s correlation coefficient R = 0.98, with positive normality test (Shapiro–Wilk) for *p*-value = 0.0273); however, the values of T_0.5_^mod^ are 1.2–5.4 times higher than T_0.5_^BOD^ determined from the BOD analysis of the same superabsorbents in our previous study [13]. On average, as shown by the slope of the upper linear trend in Figure 3a, the CO_2_ estimate gives the half-life of materials 2.1 times higher. We hypothesized two reasons for this discrepancy. First, the duration of the BOD analysis is 10–30 times longer, and it is possible that the kinetic curves of the CO_2_ analysis with a quasi-linear final section (Figure 1 and Figure 2) would change their shape with a longer incubation, with corresponding consequences for calculating the T_0.5_^mod^ indicator. Although, the assumption of an acceleration of the rate of decomposition of materials at longer stages of incubation, which is necessary to obtain lower values of T_0.5_^mod^ close to the results of the BOD analysis, seems unlikely.

The second hypothesis is the interfacial interactions of the gases studied in incubation experiments [21]. Carbon dioxide has 20–30 times and in alkaline solutions up to 1000 times greater solubility than oxygen. This means that there is a possibility of accumulation of a certain part of the CO_2_ released during the decomposition of the material in the gel structure itself. Since this amount is not taken into account by direct monitoring of CO_2_ in the air space of the incubator, there may be a strong underestimation of the rate of biodegradation of gels and, accordingly, an overestimation of their half-lives compared to the BOD analysis. To account for the immobilized carbon dioxide, we used its degassing from the gel structure by rapid heating, according to [21]. The corresponding estimate of the half-life (*T*_0.5_^term^) gave significantly smaller values (Table 3, Figure 3a lower graph). They also correlated well with the results of the BOD assessment (Pearson’s correlation coefficient R = 0.97). However, unlike the traditional CO_2_ analysis, the new methodology, taking into account the interfacial interactions of CO_2_, gave half-life values close to the results of the BOD analysis. The values of *T*_0.5_^term^ were 0.6–3.7 *T*_0.5_^BOD^ with an average linear trend coefficient of 0.84 ± 0.05 (Figure 3a lower graph), which did not statistically significantly differ from 1 at the *p*-value level of 0.001, that is, the *T*_0.5_^BOD^ and *T*_0.5_^term^ values did not differ significantly, confirming the convergence of the results of the BOD analysis and the new methodology.

To substantiate the hypothesis of interfacial interactions, we calculated from the experimental data the dimensionless Henry constants (*K*_H_^ex^) for the solubility of CO_2_ in gel structures in accordance with Henry’s law: *C*_gel_ = *K*_H_ *C*_gas_, where *C*_gas_ and *C*_gel_ are the concentrations of CO_2_ in the incubator atmosphere and in the liquid phase of the gel structure. The application of this law, taking into account the material balance in an incubator with an air volume of 200 mL and a gel structure volume of 40 mL, gives a simple formula for calculating Henry constants based on data on CO_2_ concentrations in the incubator atmosphere before and after thermo-desorption:

(9) $$K_{H}{}^{\mathrm{ex}}=\frac{200}{40}\left(\frac{C_{\mathrm{term}}}{C_{\max}}-1\right)$$

The Henry constants obtained in this way for the studied superabsorbents varied from 2 to 9.8 with an average value of 5.2 and a confidence interval of 1.8 for *p*-value = 0.01. They significantly exceed the table value of CO_2_ solubility in pure water at 30 °C (*a*_tabl_ = 0.665). However, this estimate does not take into account the formation of carbonic acid and its dissociation into bicarbonate and carbonate anions during the dissolution of CO_2_. Accounting for this most important geochemical reaction for natural aqueous solutions gives the following formula for calculating the effective solubility (Henry constant) of carbon dioxide [55]:

(10) $$K_{H}{}^{\mathrm{cal}}=a_{\mathrm{tabl}}\left(1+\frac{K_{1}}{10^{-pH}}+\frac{K_{1}K_{2}}{10^{-2pH}}\right)$$

where *K*_1,2_ are the dissociation constants of carbonic acid in the first and second stages depending on temperature, and *pH* is the indicator of the activity of hydrogen ions in an aqueous solution. In acidic solutions (*pH* < 6), *K*_H_^cal^ practically does not differ from *a*_tabl_. However, for neutral and alkaline solutions, the effective solubility *K*_H_^cal^ can significantly exceed *a*_tab_, reaching 39.4 at *pH* = 8, according to [56]. Experimental confirmation of the increase in the solubility of CO_2_ in accordance with the theoretical Equation (10) for polymer matrices on the example of casein is found in [57].

Calculated by Equation (10), the effective solubility of CO_2_ at experimental *pH* values from 6.7 to 7.4 and constants *K*_1_ = 0.51·10*^–^*^6^ and *K*_2_ = 0.45·10*^–^*^10^ gave a range of *K*_H_^cal^ from 2.4 to 9.6 with an average value of 5.4 ± 1.9 for *p*-value 0.01. Their comparison with the experimentally determined indicators *K*_H_^ex^ showed the complete identity of both values (Figure 3b). The angular coefficient of the linear trend 1.03 ± 0.02 did not statistically significantly differ from 1 for *p*-value 0.0001, which confirmed the complete identity of *K*_H_^ex^ and *K*_H_^cal^, and, accordingly, our hypothesis about the significance of CO_2_ solubility in assessing the biodegradability of gel structures by CO_2_ emission.

## 5. Conclusions

A methodological study of the biodegradability of polymer superabsorbents for soil conditioning was carried out on PASCO equipment for automated control of CO_2_ emissions during the incubation of organic materials. The new physically based models developed in the study made it possible to adequately describe the typical forms of the kinetic curves of the biodegradation of superabsorbents and estimate their half-lives. The revealed strong (up to 10 times) decrease in the half-lives of synthetic hydrogels under the influence of compost extract calls into question the traditional opinion about these materials as “not biodegradable”. Apparently, not only the chemical composition and structure of polymer hydrogels but also the presence or absence of specific microflora and its exoenzymes determine the stability and service life of soil water superabsorbents under real environmental conditions. The introduction of silver ions at a dose of 0.1% into the acrylic polymer matrix of gel-forming soil conditioners is an effective means of controlling their resistance to biodegradation, increasing it to the European standard for the resistance of synthetic polymers in soils with a half-life of more than 6 years. However, the relative high cost of silver biocides requires a future search for equally effective but less costly biodegradability controls for gel-forming soil conditioners. The methodologies for laboratory evaluation of biodegradability using CO_2_ emission and biological oxygen demand correlate well with each other but can differ greatly (up to two times or more) in the absolute values of the half-lives of the studied materials. The most likely reason for these differences is the lack of allowance for the dissolution of carbon dioxide in the liquid phase of gel structures in the traditional CO_2_ analysis. Correction for interfacial interactions of carbon dioxide, reconciling both methodologies, can be carried out either experimentally by CO_2_ thermal degassing or by calculation using effective Henry’s constants depending on the temperature and *pH* of the liquid phase in the gel structure.

## 6. Patents

The results of this work are used in the synthesis technology of biodegradation-resistant filled hydrogels patented in the Russian Federation:

- patent RU 2726561 (https://findpatent.ru/patent/272/2726561.html, accessed on 22 May 2023);
- patent RU 2639789 (http://www.findpatent.ru/patent/263/2639789.html, accessed on 22 May 2022).

## Figures and Tables

**Table 1:** Composition and some properties of gel-forming soil conditioners.

Superabsorbent	Composition	*C*%	*W_h_*%	pH
Aquasorb	AcK, PAA *,	39.5 ± 0.5	32 ± 3	7.3 ± 0.1
Zeba	PAA, AcK, starch	46.6 ± 0.7	11 ± 5	7.0 ± 0.1
A11	AcAm, PAA, PAA-biocatalysis waste	45.0 ± 0.6	30 ± 2	7.2 ± 0.1
A11-Ag	AcAm, PAA, PAA-biocatalysis waste, silver	45.0 ± 0.4	30 ± 4	7.3 ± 0.1
A22	AcNa, PAA, peat	47.5 ± 0.5	31 ± 2	7.4 ± 0.1
A22-Ag	AcNa, PAA, peat, silver	47.5 ± 0.3	35 ± 3	7.4 ± 0.1

* Denotation: PAA—polyacrylamide; AcK, AcNa, and AcAm—potassium, sodium, and ammonium acrylates; for swollen 1:100 gels in pure distilled water; here and below ± is the confidence interval.

**Table 2:** Parameters and statistical indicators for the approximation of CO_2_ kinetic curves by models (1) and (2).

Approximation Parameters for Models (1) and (2):			Statistical Indicators:		
*a*	*b* or *r*	*x*_0_	*p-*Value	*R* ^2^	*s*
Aquasorb–W *					
0.618 ± 0.002	68.51 ± 1.86	–	<0.0001	0.956	0.028
Aquasorb–Cex					
0.789 ± 0.005	8.97 ± 0.09	0.564 ± 0.001	<0.0001	0.998	0.019
Zeba–W					
0.0008 ± 0.0001	0.0012 ± 0.0001	–	0.0013	0.999	0.010
Zeba–Cex					
0.547 ± 0.003	11.56 ± 0.19	0.422 ± 0.001	<0.0001	0.998	0.015
A11–W					
0.801 ± 0.002	10.84 ± 0.11	–	<0.0001	0.987	0.022
A11–Ag–W					
0.799 ± 0.005	9.56 ± 0.18	–	<0.0001	0.965	0.037
A11–Cex					
0.559 ± 0.007	9.35 ± 0.14	0.522 ± 0.001	<0.0001	0.996	0.024
A11–Ag–Cex					
0.768 ± 0.004	5.93 ± 0.14	–	<0.0001	0.981	0.028
A22–W					
0.499 ± 0.005	19.90 ± 0.89	–	<0.0001	0.968	0.035
A22–Ag–W					
0.589 ± 0.007	14.55 ± 0.64	–	<0.0001	0.955	0.041
A22–Cex					
0.397 ± 0.005	13.81 ± 0.42	0.646 ± 0.001	<0.0001	0.997	0.019
A22–Ag–Cex					
0.621 ± 0.017	4.83 ± 0.19	–	<0.0001	0.991	0.023

* Denotation: W, Cex—gels swollen in pure distilled water and with the addition of compost extract, respectively.

**Table 3:** Scaling parameters of CO_2_ emission kinetic curves and estimation of half-lives for gel-forming superabsorbents.

Scaling Parameters:			Half-Lives, Years:		
*C*_max_, g/m^3^	*C*_t_ *, g/m^3^	*T*_max_, hr	*T*_0.5_^min^–*T*_0.5_^fin^	*T*_0.5_^mod^	*T*_0.5_^term^
Aquasorb–W					
9.6	23.3	71.8	1.91–3.53	3.40 ± 0.04	1.40 ± 0.02
Aquasorb–Cex					
103.0	147.8	71.8	0.12–0.45	0.58 ± 0.01	0.40 ± 0.01
Zeba–W					
27.6	50.9	62.8	0.59–0.59	0.56 ± 0.07	0.32 ± 0.05
Zeba–Cex					
134.0	210.1	59.0	0.11–0.23	0.24 ± 0.04	0.15 ± 0.02
A11–W					
20.4	43.2	64.4	0.67–2.71	3.17 ± 0.06	1.50 ± 0.03
A11-Ag–W					
3.6	8.8	50.2	3.18–10.86	13.84 ± 0.48	5.70 ± 0.20
A11–Cec					
135.0	188.6	55.5	0.08–0.14	0.18 ± 0.03	0.13 ± 0.02
A11–Ag–Cex					
22.0	42.6	60.0	0.59–2.76	2.38 ± 0.06	1.22 ± 0.03
A22–W					
1.8	4.8	23.7	3.48–6.07	5.49 ± 0.10	2.07 ± 0.05
A22–Ag–W					
2.4	7.1	60.0	6.64–13.90	12.26 ± 0.34	4.20 ± 0.12
A22–Cex					
54.0	99.2	48.0	0.19–0.26	0.31 ± 0.05	0.17 ± 0.02
A22–Ag–Cex					
16.0	31.3	83.0	1.41–3.37	3.38 ± 0.17	1.68 ± 0.08

* Denotation: *C*_max_, *C*_t_—final CO_2_ content in the incubator before and after thermo-desorption; *T*_0.5_^mod^, *T*_0.5_^term^—half-life estimation by models (1) and (2) before and after CO_2_ thermo-desorption; for other designations, see Table 2.

**Table 4:** Probability matrix (*p*-values) for post hoc group comparisons.

Gels:	AQ p. *	AQ e.	ZB p.	ZB e.	A11 p.	A11-Ag p.	A11 e.	A11-Ag e.	A22 p.	A22-Ag p.	A22 e.	A22-Ag e.
Parametric ANOVA (LSD test)												
AQ p.		1.5 × 10^−2^	1.9 × 10^−2^	8.2 × 10^−3^	6.1 × 10^−1^	1.0 × 10^−9^	7.0 × 10^−3^	3.3 × 10^−1^	5.5 × 10^−2^	3.2 × 10^−10^	9.6 × 10^−3^	8.9 × 10^−1^
AQ e.	1.5 × 10^−2^		9.2 × 10^−1^	8.1 × 10^−1^	5.1 × 10^−2^	2.0 × 10^−13^	7.7 × 10^−1^	1.3 × 10^−1^	4.5 × 10^−5^	6.7 × 10^−14^	8.6 × 10^−1^	2.1 × 10^−2^
ZB p.	1.9 × 10^−2^	9.2 × 10^−1^		7.3 × 10^−1^	6.4 × 10^−2^	2.8 × 10^−13^	6.9 × 10^−1^	1.6 × 10^−1^	6.3 × 10^−5^	9.3 × 10^−14^	7.8 × 10^−1^	2.7 × 10^−2^
ZB e.	8.2 × 10^−3^	8.1 × 10^−1^	7.3 × 10^−1^		3.0 × 10^−2^	9.1 × 10^−14^	9.5 × 10^−1^	8.1 × 10^−2^	2.0 × 10^−5^	3.1 × 10^−14^	9.5 × 10^−1^	1.2 × 10^−2^
A11 p.	6.1 × 10^−1^	5.1 × 10^−2^	6.4 × 10^−2^	3.0 × 10^−2^		1.7 × 10^−10^	2.6 × 10^−2^	6.5 × 10^−1^	1.6 × 10^−2^	5.3 × 10^−11^	3.4 × 10^−2^	7.0 × 10^−1^
A11-Ag p.	1.0 × 10^−9^	2.0 × 10^−13^	2.8 × 10^−13^	9.1 × 10^−14^	1.7 × 10^−10^		7.6 × 10^−14^	3.5 × 10^−11^	1.1 × 10^−6^	7.4 × 10^−1^	1.1 × 10^−13^	6.5 × 10^−10^
A11 e.	7.0 × 10^−3^	7.7 × 10^−1^	6.9 × 10^−1^	9.5 × 10^−1^	2.6 × 10^−2^	7.6 × 10^−14^		7.2 × 10^−2^	1.7 × 10^−5^	2.6 × 10^−14^	9.1 × 10^−1^	1.0 × 10^−2^
A11-Ag e.	3.3 × 10^−1^	1.3 × 10^−1^	1.6 × 10^−1^	8.1 × 10^−2^	6.5 × 10^−1^	3.5 × 10^−11^	7.2 × 10^−2^		5.0 × 10^−3^	1.1 × 10^−11^	9.1 × 10^−2^	4.1 × 10^−1^
A22 p.	5.5 × 10^−2^	4.5 × 10^−5^	6.3 × 10^−5^	2.0 × 10^−5^	1.6 × 10^−2^	1.1 × 10^−6^	1.7 × 10^−5^	5.0 × 10^−3^		3.3 × 10^−7^	2.5 × 10^−5^	4.1 × 10^−2^
A22-Ag p.	3.2 × 10^−10^	6.7 × 10^−14^	9.3 × 10^−14^	3.1 × 10^−14^	5.3 × 10^−11^	7.4 × 10^−1^	2.6 × 10^−14^	1.1 × 10^−11^	3.3 × 10^−7^		3.8 × 10^−14^	2.0 × 10^−10^
A22 e.	9.6 × 10^−3^	8.6 × 10^−1^	7.8 × 10^−1^	9.5 × 10^−1^	3.4 × 10^−2^	1.1 × 10^−13^	9.1 × 10^−1^	9.1 × 10^−2^	2.5 × 10^−5^	3.8 × 10^−14^		1.4 × 10^−2^
A22-Ag e.	8.9 × 10^−1^	2.1 × 10^−2^	2.7 × 10^−2^	1.2 × 10^−2^	7.0 × 10^−1^	6.5 × 10^−10^	1.0 × 10^−2^	4.1 × 10^−1^	4.1 × 10^−2^	2.0 × 10^−10^	1.4 × 10^−2^	
Nonparametric ANOVA (Newman–Keuls test)												
AQ p.		1.4 × 10^−1^	1.3 × 10^−1^	1.3 × 10^−1^	8.6 × 10^−1^	1.3 × 10^−4^	1.4 × 10^−1^	7.6 × 10^−1^	5.5 × 10^−2^	1.7 × 10^−4^	1.2 × 10^−1^	8.9 × 10^−1^
AQ e.	1.4 × 10^−1^		9.2 × 10^−1^	9.7 × 10^−1^	2.0 × 10^−1^	1.3 × 10^−4^	9.9 × 10^−1^	2.8 × 10^−1^	9.6 × 10^−4^	1.4 × 10^−4^	8.6 × 10^−1^	1.4 × 10^−1^
ZB p.	1.3 × 10^−1^	9.2 × 10^−1^		9.9 × 10^−1^	1.5 × 10^−1^	1.4 × 10^−4^	9.9 × 10^−1^	1.6 × 10^−1^	9.7 × 10^−4^	1.3 × 10^−4^	9.6 × 10^−1^	1.2 × 10^−1^
ZB e.	1.3 × 10^−1^	9.7 × 10^−1^	9.9 × 10^−1^		2.4 × 10^−1^	1.5 × 10^−4^	9.6 × 10^−1^	3.9 × 10^−1^	7.5 × 10^−4^	1.8 × 10^−4^	9.5 × 10^−1^	1.4 × 10^−1^
A11 p.	8.6 × 10^−1^	2.0 × 10^−1^	1.5 × 10^−1^	2.4 × 10^−1^		1.3 × 10^−4^	2.7 × 10^−1^	6.5 × 10^−1^	7.5 × 10^−2^	1.4 × 10^−4^	2.1 × 10^−1^	7.0 × 10^−1^
A11-Ag p.	1.3 × 10^−4^	1.3 × 10^−4^	1.4 × 10^−4^	1.5 × 10^−4^	1.3 × 10^−4^		1.8 × 10^−4^	1.4 × 10^−4^	1.2 × 10^−4^	7.4 × 10^−1^	1.4 × 10^−4^	1.7 × 10^−4^
A11 e.	1.4 × 10^−1^	9.9 × 10^−1^	9.9 × 10^−1^	9.6 × 10^−1^	2.7 × 10^−1^	1.8 × 10^−4^		4.5 × 10^−1^	7.8 × 10^−4^	1.3 × 10^−4^	9.9 × 10^−1^	1.5 × 10^−1^
A11-Ag e.	7.6 × 10^−1^	2.8 × 10^−1^	1.6 × 10^−1^	3.9 × 10^−1^	6.5 × 10^−1^	1.4 × 10^−4^	4.5 × 10^−1^		3.9 × 10^−2^	1.4 × 10^−4^	3.2 × 10^−1^	6.8 × 10^−1^
A22 p.	5.5 × 10^−2^	9.6 × 10^−4^	9.7 × 10^−4^	7.5 × 10^−4^	7.5 × 10^−2^	1.2 × 10^−4^	7.8 × 10^−4^	3.9 × 10^−2^		1.3 × 10^−4^	7.3 × 10^−4^	1.0 × 10^−1^
A22-Ag p.	1.7 × 10^−4^	1.4 × 10^−4^	1.3 × 10^−4^	1.8 × 10^−4^	1.4 × 10^−4^	7.4 × 10^−1^	1.3 × 10^−4^	1.4 × 10^−4^	1.3 × 10^−4^		1.5 × 10^−4^	1.3 × 10^−4^
A22 e.	1.2 × 10^−1^	8.6 × 10^−1^	9.6 × 10^−1^	9.5 × 10^−1^	2.1 × 10^−1^	1.4 × 10^−4^	9.9 × 10^−1^	3.2 × 10^−1^	7.3 × 10^−4^	1.5 × 10^−4^		1.3 × 10^−1^
A22-Ag e.	8.9 × 10^−1^	1.4 × 10^−1^	1.2 × 10^−1^	1.4 × 10^−1^	7.0 × 10^−1^	1.7 × 10^−4^	1.5 × 10^−1^	6.8 × 10^−1^	1.0 × 10^−1^	1.3 × 10^−4^	1.3 × 10^−1^	

* Denotation: AQ—Aquasorb; ZB—Zeba; p. and e.—gels swollen in pure distilled water and with the addition of compost extract, respectively.

## Data Availability

The data presented in this study are available on request from the corresponding author. The data are not publicly available due to privacy until the end of the scientific project.
